# Case Report: Hereditary transthyretin amyloidosis in a Chinese family with the transthyretin variant p.Y89H

**DOI:** 10.3389/fneur.2026.1962236

**Published:** 2026-09-14

**Authors:** Fengzhu Zhao, Yijing Xiao, Yingjia Li, Jiangnan Yu, Jiangqin Song, Tong Lu

**Affiliations:** 1 Department of Neurology, The First People's Hospital of Tianmen City in Hubei Province, Tianmen, China; 2 School of Medicine, Wuhan University of Science and Technology, Wuhan, Hubei, China; 3 Department of Urinary Surgery, The First People’s Hospital of Tianmen (Tianmen Hospital Affiliated to Wuhan University of Science and Technology), Tianmen, Hubei, China; 4 Department of Gastroenterology, The First People's Hospital of Tianmen City in Hubei Province, Tianmen, China; 5 Department of Neurology, Union Hospital, Tongji Medical College, Huazhong University of Science and Technology, Wuhan, China; 6 Department of Medical Laboratory, The First People’s Hospital of Tianmen (Tianmen Hospital Affiliated to Wuhan University of Science and Technology), Tianmen, Hubei, China; 7 Hubei Province Key Laboratory of Occupational Hazard Identification and Control, Wuhan University of Science and Technology, Wuhan, Hubei, China

**Keywords:** case report, hereditary transthyretin amyloidosis, leptomeningeal amyloidosis, p.Y89H, visual decline

## Abstract

**Background:**

Hereditary transthyretin amyloidosis (hATTR) is a rare, life-threatening multisystem disorder caused by pathogenic variants in the transthyretin (TTR) gene. Its clinical manifestations are highly heterogeneous, frequently resulting in diagnostic delay or misdiagnosis.hATTR caused by the TTR p.Y89H variant is extremely rare and is associated with a phenotype characterized by predominant ocular and leptomeningeal involvement.

**Case presentation:**

A 59-year-old woman presented with a 10-year history of episodic headaches and a 3-year history of progressive bilateral visual decline. Brain computed tomography (CT) showed persistent diffuse leptomeningeal hyperdensities, and contrast-enhanced magnetic resonance imaging (MRI) revealed extensive, homogeneous enhancement of the cerebral and spinal leptomeninges. Genetic testing identified a heterozygous TTR variant, c.265 T > C (p.Y89H), in both the patient and her younger brother. The patient underwent left vitrectomy for vitreous opacities and received symptomatic treatment with ibuprofen for episodic headaches. Visual acuity in the left eye improved after vitrectomy, whereas visual acuity in the right eye continued to decline progressively during follow-up. CT obtained at one-year follow-up showed persistent leptomeningeal hyperdensities.

**Conclusion:**

In the appropriate clinical and genetic context, persistent leptomeningeal hyperdensities on brain CT may serve as a diagnostic clue for p.Y89H-associated hATTR, although this finding should be interpreted cautiously and differentiated from other leptomeningeal pathologies. Patients with leptomeningeal hATTR may also be susceptible to contrast-induced encephalopathy.

## Introduction

Hereditary transthyretin amyloidosis (hATTR) is a rare and life-threatening multisystem disease caused by pathogenic variants in the transthyretin (TTR) gene and inherited in an autosomal dominant pattern. It is characterized predominantly by chronic peripheral neuropathy and cardiac involvement; in rare cases, the eyes and leptomeninges are predominantly affected, a phenotype referred to as oculoleptomeningeal amyloidosis (1). To date, more than 140 amyloidogenic TTR variants have been reported worldwide (2). hATTR arising from the TTR p.Y89H variant is extremely rare. We herein describe a case of hATTR associated with the p.Y89H variant in a Chinese woman, including an analysis of her clinical manifestations and imaging findings. Our objective is to share the diagnostic experience and analyze the clinical features of comparable cases, thereby reducing misdiagnosis.

## Case presentation

A 59-year-old woman was admitted to our hospital with a 10-year history of headaches and a 3-year history of decreased visual acuity in both eyes. Ten years earlier, she began experiencing episodic headaches without identifiable triggers, characterized by severe bursting pain in the bilateral frontal and temporal regions. The headaches were not throbbing and were not accompanied by nausea or vomiting. Each episode lasted approximately 10–20 min and occurred 3–5 times annually. Three years earlier, she developed asymmetric visual decline in both eyes, with the left eye more severely affected. She also reported mild memory decline but no limb weakness or speech difficulties. One week before admission, she was scheduled for ophthalmic surgery because of vitreous opacities. However, a preoperative head computed tomography (CT) scan suggested possible subarachnoid hemorrhage (SAH), leading to her transfer to our hospital. The patient had no cardiac symptoms and no history of hypertension, diabetes, or cardiac disease. She denied any family history of similar conditions and had no prior history of surgery, trauma, allergies, or toxic exposure. She had not taken any long-term medications.

On examination, the patient’s blood pressure was 114/73 mmHg. Her neurological examination findings were unremarkable, and no clinical evidence of peripheral nerve involvement was observed. Electrocardiography, serum cardiac troponin, and transthoracic echocardiography were normal. Given the absence of cardiac symptoms and the normal noninvasive cardiac evaluation, B-type natriuretic peptide (BNP) testing and cardiac biopsy were not performed. Ophthalmologic evaluation revealed vitreous opacities, with the left eye more severely affected. Her visual acuity was 20/80 in the left eye and 20/25 in the right eye. Brain CT showed diffuse leptomeningeal hyperdensity, predominantly around the bilateral sylvian fissures, with attenuation values exceeding 100 Hounsfield units (HU) (Figure 1A). Lumbar puncture demonstrated clear cerebrospinal fluid (CSF) with normal opening pressure. CSF analysis was notable only for an elevated protein level of 1.117 g/L (reference range, 0.15–0.45 g/L), whereas the white blood cell count, red blood cell count, and other parameters were within normal ranges. Computed tomography angiography (CTA) of the head and neck vessels was negative. T1-weighted and T2-weighted brain images showed no significant abnormalities (Figures 1B,C). However, sagittal contrast-enhanced T1-weighted images revealed extensive and homogeneous enhancement of the cerebral and spinal leptomeninges (Figures 1D,E). Susceptibility-weighted imaging (SWI) was unremarkable (Figure 1F).

**Figure 1:**
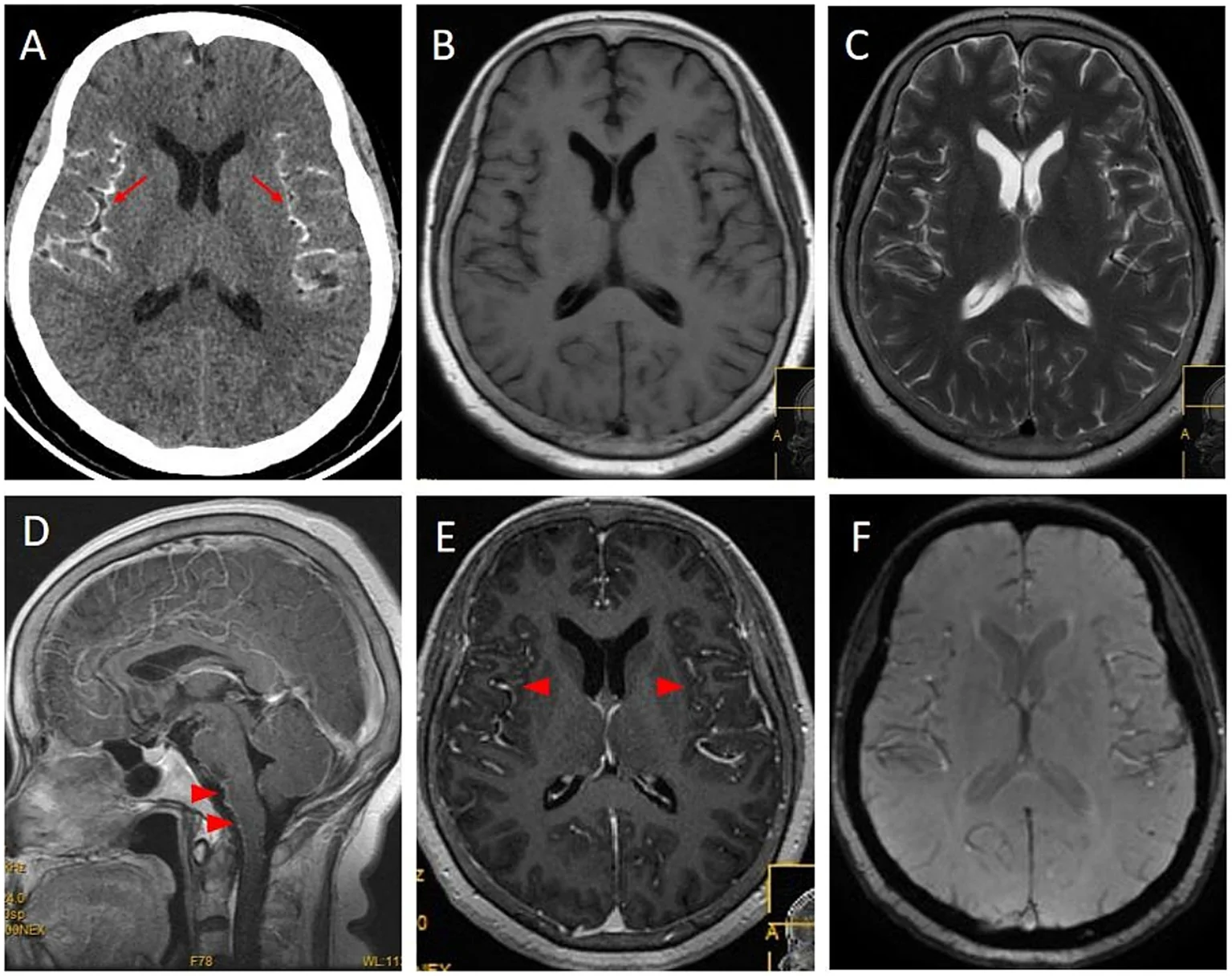
Neuroimaging findings in the proband with p. Y89H-associated hereditary transthyretin amyloidosis. **(A)** Axial computed tomography scan demonstrates diffuse leptomeningeal hyperdensity, predominantly around the bilateral sylvian fissures (red arrows), with attenuation values exceeding 100 Hounsfield units. **(B,C)** Axial T1-weighted and T2-weighted images show no significant abnormalities. **(D,E)** Sagittal and axial contrast-enhanced T1-weighted images reveal extensive and homogeneous enhancement of the cerebral and spinal leptomeninges (red arrowheads). **(F)** Susceptibility-weighted imaging shows no hemorrhage.

One year later, her younger brother presented to our hospital with transient focal neurological episodes (TFNEs) and tremor, and he developed encephalopathy after cerebral angiography. His CSF protein level was 1.46 g/L with a normal cell count, and his neuroimaging findings closely resembled those of the proband. Tissue biopsy was recommended but was declined by the patient and her family members.

### Genetic investigation

The same heterozygous missense variant in the TTR gene, c.265 T > C (p.Y89H) (Figure 2), was identified in both the patient and her younger brother. According to the American College of Medical Genetics and Genomics/Association for Molecular Pathology (ACMG/AMP) criteria, this variant was classified as pathogenic, supported by PP1_Strong, PM1, PS4_Moderate, PP3_Moderate, PS3_Supporting, and PM2_Supporting. Therefore, the diagnosis of hATTR was established based on ocular and leptomeningeal involvement, CSF protein elevation with a normal cell count, diffuse leptomeningeal enhancement, family history, and genetic evidence. Additionally, the patient’s mother and elder sister carried the TTR p.Y89H variant but were asymptomatic.

**Figure 2:**
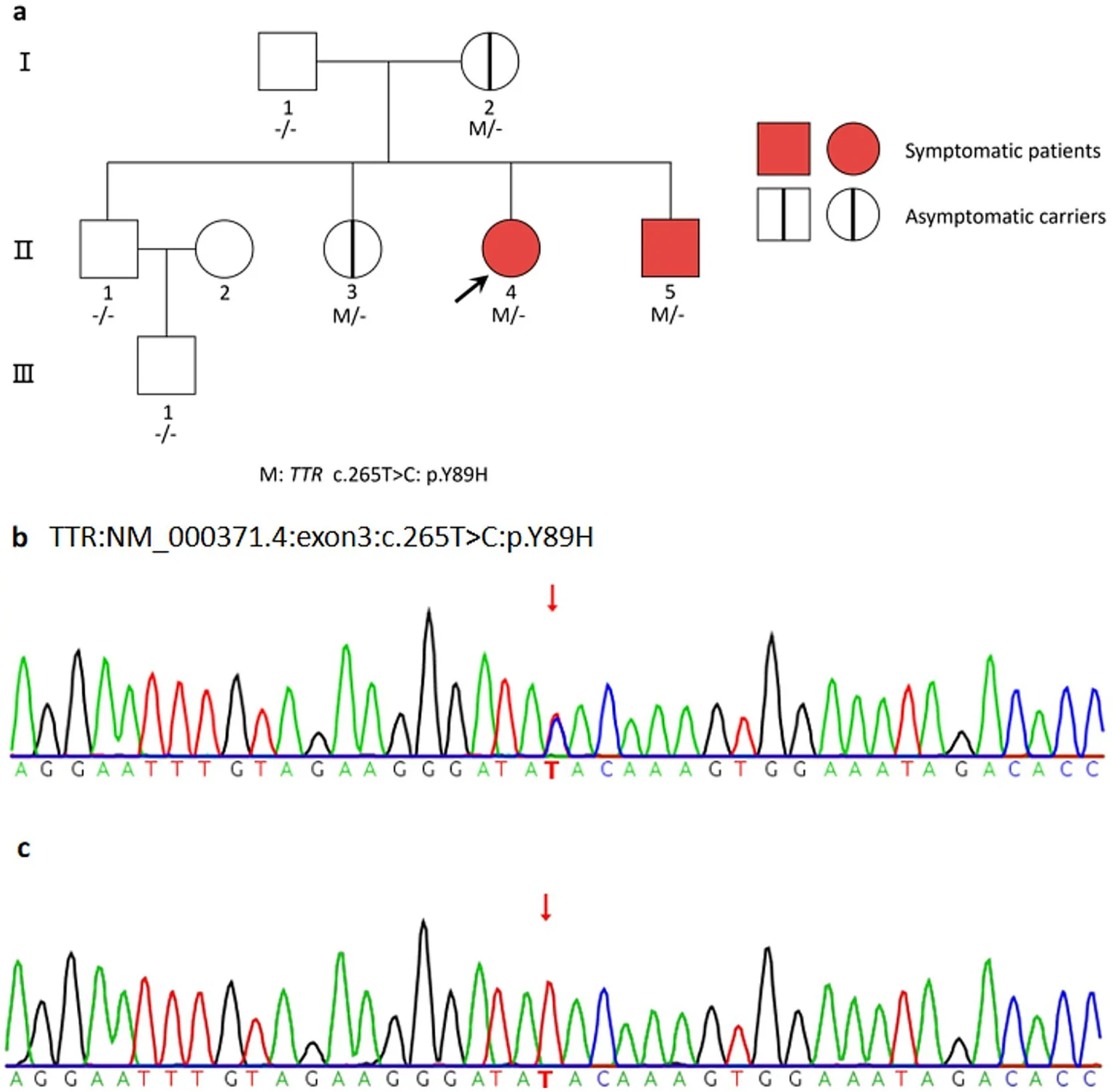
Pedigree and sequencing results of the family carrying the heterozygous Transthyretin (TTR) variant c.265 T > C (p.Y89H). Squares and circles indicate male and female family members, respectively; filled red symbols indicate variant carriers with corresponding clinical symptoms or abnormal examinations, half-filled symbols indicate asymptomatic variant carriers, and open symbols indicate relatives without the detected variant. The proband, her younger brother, elder sister, and mother carried the heterozygous missense variant, whereas the proband’s father, elder brother, and nephew had wild-type TTR sequences **(a)**. Genomic DNA sequencing confirmed the heterozygous TTR variant c.265 T > C (p.Y89H) in the proband (II:4) **(b)** compared with the wild-type sequence in an unaffected family member (I:1) **(c)**.

### Outcome and follow-up

The patient underwent vitrectomy of the left eye, resulting in significant improvement in visual acuity. Her headaches were relieved by ibuprofen during episodes. One-year follow-up brain CT demonstrated persistent diffuse leptomeningeal hyperdensities. During continued follow-up, the patient’s visual acuity in the right eye progressively worsened. She also reported intermittent headaches and mild cognitive decline.

## Discussion

hATTR is a rare and life-threatening multisystem disease caused by pathogenic variants in the TTR gene and inherited in an autosomal dominant pattern. Oculoleptomeningeal amyloidosis represents a rare subtype of hATTR, characterized by predominant deposition of abnormal TTR in the leptomeninges and vitreous body (3).

A systematic search of PubMed was conducted to identify reported cases carrying the p.Y89H variant between January 1980 and February 2026, using the following query: (Tyr69His) OR (Y69H) OR (Tyr89His) OR (Y89H) (3–9). No language restriction was applied, and non-English publications were considered, including a Japanese-language report (7). Reports were included if they described individual patients or pedigrees with a genetically confirmed TTR p.Y89H/Tyr69His/Y69H variant and extractable clinical, imaging, cerebrospinal fluid, pathological, or systemic evaluation data. Reviews without original patient-level data, non-human studies, reports without extractable individual-level information, and duplicate descriptions of the same individuals were excluded. When multiple reports described the same pedigree, overlapping individuals were assessed and counted only once. Overall, 12 previously reported hATTR cases with the p.Y89H variant were identified worldwide (Table 1) (3–9). As three of the previously reported cases originated from a single Swedish pedigree, we carefully assessed and excluded the possibility of overlapping patients in our analysis. The age at onset of these reported cases ranged from 32 to 68 years. Most patients (9/12, 75.0%) showed leptomeningeal involvement. Mild peripheral neuropathy was present in five patients (5/12, 41.7%), and four patients (4/12, 33.3%) developed severe ocular manifestations. The most common peripheral nerve symptom was bilateral carpal tunnel syndrome (3/12, 25.0%). No clinical evidence of cardiac or renal involvement was observed in the summarized cases; however, myocardial biopsy in some patients revealed amyloid deposits, indicating possible subclinical cardiac involvement (3).

**Table 1:** Characteristics of 12 previously reported patients and two symptomatic members of the present family carrying the p.Y89H variant in the transthyretin gene.

From	NO	Sex	Age at onset, years	Clinical phenotype	CSF protein (g/L)	CSF cell	MRI (diffuse leptomeningeal enhancement)	Pathological examination (amyloid)	Other examinations
Blevins et al. 2003 (3)	1	F	68	Stroke-like episodes, mild ataxia, mild peripheral axonal neuropathy, encephalopathy after Carotid angiogram	/	/	+	Skin biopsy (−)	NCV (+), Renal (−) Cardiac (−), Eye (−),
	2	M	40	Seizures, stroke-like episodes, vitreous amyloid, encephalopathy after Cardiac angiogram	0.85	−	+	Skin and cardiac (+)	NCV (−), Renal (−)
	3	F	47	Vitreous amyloid, episodic dysarthria	/	/	+	Skin biopsy (−), vitreous sample (+)	NCV (−), Renal (−), Cardiac (−)
	4	F	37	Radiculopathy	/	/	−	Skin biopsy (−)	Eye (−), NCV (−), Renal (−), Cardiac (−)
	5	M	39	Normal	/	/	−	Skin biopsy (−)	Eye (−), NCV (−)
Schweitzer et al. 2009 (4)	6	M	59	Vitreous amyloid, transient events (disorientation, memory loss, confusion, expressive aphasia, numbness, nausea, emesis, bifrontal headaches, and fatigue)	/	/	+	Vitreous sample (+)	/
Suhr et al. 2009 (5)	7	F	52	Vitreous amyloid, stroke-like episodes, bilateral carpal tunnel syndrome, seizure, dementia	/	/	+	Fat and skin (+)	Holter ECG (−), UCG (−)
Yamada et al. 2019 (6)	8	M	41	Bilateral carpal tunnel syndrome, nausea and dizziness, diminished consciousness, bout of syncope, TFNEs	↑	↑	+	Pia mater and subpial area (+)	NCV (−), ECG (−), EEG (−)
Saito et al. 2023 (7)	9	F	66	TFNEs, ataxia, cognitive impairment, drop attacks and recurrent transient language disorder	1.57	−	+	Subarachnoid vessel (+)	NCV (+), ECG (−), Eye (−)
Ziskin et al. 2015 (8)	10	M	59	Progressive cognitive decline, multiple encephalopathic episodes (headache, confusion, ataxia and short-term memory loss)	/	/	/	Meningovascular and ventricular (+)	/
Quintanilha et al. 2020 (9)	11	M	33	Stroke-like episodes, tension type headache, mental confusion, aphasia, seizure after cerebral arteriography	0.6	−	+	Arachnoid and superficial cerebral cortex (+)	EEG (asymmetric polymorphic delta waves)
	12	F	32	Mild orthostatic hypotension, sensory small fiber neuropathy, bilateral carpal tunnel syndrome	/	/	−	fat biopsy (−)	EEG (−), ECG (−), Eye (−)
Present Case (proband)	13	F	49	Episodic headaches, vitreous opacities with visual decline, mild cognitive decline, persistent leptomeningeal hyperdensities and enhancement	1.117	−	+	Tissue biopsy refused	Cardiac symptoms (−), peripheral neuropathy (−), ECG (−), UCG (−), troponin (−), BNP not tested, CTA (−), SWI (−), genetic testing (+)
Present Case (younger brother)	14	M	50	TFNEs, encephalopathy after cerebral angiography, tremor	1.46	−	+	Tissue biopsy refused	Genetic testing (+)

The two symptomatic members of the present family are highlighted in red shading and were not included in the percentage calculations for previously reported cases. Asymptomatic variant carriers shown in the pedigree are not listed in this table. CSF, cerebrospinal fluid; MRI, magnetic resonance imaging; CTA, computed tomography angiography; SWI, susceptibility-weighted imaging; TFNEs, transient focal neurological episodes; NCV, nerve conduction velocity; UCG, echocardiography; ECG, electrocardiogram; EEG, electroencephalogram; BNP, B-type natriuretic peptide; /, not provided or not applicable; +, positive; −, negative; upward arrow, elevated; F, female; M, male.

The clinical spectrum of p.Y89H-associated hATTR appears variable, even within the same family. In the present pedigree, the proband and her younger brother had neurological, ocular, and leptomeningeal manifestations, whereas the mother and elder sister carried the same variant but were asymptomatic at the time of evaluation. The asymptomatic carriers received genetic counseling, and head CT was normal in all asymptomatic carriers. This intrafamilial variability is consistent with incomplete or age-dependent penetrance and supports genetic counseling, cascade testing, and longitudinal surveillance of asymptomatic carriers, particularly for neurological, ophthalmologic, cardiac, renal, and autonomic manifestations.

Early manifestations in patients with the p.Y89H variant often include TFNEs, seizures, and visual decline. Headaches may occur independently, presenting as transient tension-type headaches or bilateral frontal pain, possibly related to cortical spreading depression (CSD) induced by leptomeningeal pathology (6). Severe cognitive deterioration, ataxia, and tremor may appear in advanced disease. In addition to leptomeningeal involvement, ocular damage in p.Y89H variant carriers can be severe, leading to substantial visual impairment. Vitreous opacities and retinal lesions may be detected on ophthalmologic examination, and vitrectomy has been shown to improve visual acuity (4). The improvement in left-eye visual acuity after vitrectomy in our patient is consistent with findings reported in the literature.

Notably, a quarter of the 12 previously reported patients with the p.Y89H variant presented with encephalopathy or seizures after the injection of contrast agents, commonly referred to as contrast-induced encephalopathy (CIE) (3, 9). These observations suggest that TTR deposition in the leptomeninges may be associated with chronic leptomeningeal inflammation and increased vascular permeability, which could facilitate leakage of hyperosmolar contrast agents into the subarachnoid space. Therefore, iodinated contrast agents and other hyperosmolar agents should be used cautiously in hATTR patients with leptomeningeal involvement.

Most reported patients with the p.Y89H variant had elevated CSF protein levels and normal or mildly increased cell counts. Previous imaging studies of leptomeningeal involvement in patients with the p.Y89H variant have primarily focused on brain MRI. Contrast-enhanced brain MRI revealed extensive and homogeneous enhancement of the cerebral and spinal pia mater in most patients (8/12, 66.7%). CT findings were rarely reported but may provide useful diagnostic clues in the appropriate clinical and genetic context. Reported CT abnormalities include persistent diffuse hyperdense foci along the leptomeningeal surface, with attenuation values exceeding those typically expected for hemorrhage. However, similar leptomeningeal hyperdensities or calcification-like changes may also occur in other leptomeningeal disorders, prior hemorrhage, inflammatory disease, infection, or metabolic conditions; therefore, CT findings should not be considered diagnostic in isolation. Another study reported diffuse calcification around the brainstem and spinal cord in a patient with hATTR. The calcifications were potentially attributed to aseptic meningitis or recurrent SAH related to amyloid-associated vascular fragility (10). Given that normal SWI in our patient largely ruled out hemorrhage and that the leptomeningeal hyperdensities detected on CT scan were not visible on MRI, we speculate that these hyperdensities resulted from a mixture of calcium salts and amyloid deposits.

Current therapeutic options for hATTR include symptomatic management, liver transplantation, and targeted pharmacotherapy. Targeted medications include TTR stabilizers, such as tafamidis, and gene-silencing agents, such as vutrisiran (11). However, the efficacy of these drugs in preventing disease progression remains uncertain in patients with the p.Y89H variant because clinical experience and variant-specific evidence remain limited. Vitrectomy has been shown to improve visual impairment caused by vitreous opacities.

In summary, hATTR caused by the p.Y89H variant mainly involves the leptomeninges and eyes. Brain CT may reveal persistent diffuse leptomeningeal hyperdensities, which can provide an important diagnostic clue when interpreted together with clinical features, MRI findings, CSF abnormalities, family history, and genetic testing. Importantly, patients with leptomeningeal amyloidosis may be more susceptible to contrast-induced encephalopathy.

## Data Availability

The original contributions presented in the study are included in the article/supplementary material, further inquiries can be directed to the corresponding author.
